# Genotyping by Genome Reducing and Sequencing for Outbred Animals

**DOI:** 10.1371/journal.pone.0067500

**Published:** 2013-07-18

**Authors:** Qiang Chen, Yufang Ma, Yumei Yang, Zhenliang Chen, Rongrong Liao, Xiaoxian Xie, Zhen Wang, Pengfei He, Yingying Tu, Xiangzhe Zhang, Changsuo Yang, Hongjie Yang, Fuqing Yu, Youmin Zheng, Zhiwu Zhang, Qishan Wang, Yuchun Pan

**Affiliations:** 1 School of Agriculture and Biology, Shanghai Jiao Tong University, Shanghai, China; 2 Jiangsu Normal University, Xuzhou, China; 3 Shanhai Genome Biotechnology Company, Shanghai, China; 4 National Research Centre on Poultry Engineering Technology, Shanghai Academy of Agricultural Science, Shanghai, China; 5 National Animal Husbandry Services, Beijng, China; 6 Institute for Genomic Diversity, Cornell University, Ithaca, New York, United States of America; 7 Shanghai Key Laboratory of Veterinary Biotechology, Shanghai, China; Huazhong Agricultural University, China

## Abstract

Next-generation sequencing (NGS) approaches are widely used in genome-wide genetic marker discovery and genotyping. However, current NGS approaches are not easy to apply to general outbred populations (human and some major farm animals) for SNP identification because of the high level of heterogeneity and phase ambiguity in the haplotype. Here, we reported a new method for SNP genotyping, called genotyping by genome reducing and sequencing (GGRS) to genotype outbred species. Through an improved procedure for library preparation and a marker discovery and genotyping pipeline, the GGRS approach can genotype outbred species cost-effectively and high-reproducibly. We also evaluated the efficiency and accuracy of our approach for high-density SNP discovery and genotyping in a large genome pig species (2.8 Gb), for which more than 70,000 single nucleotide polymorphisms (SNPs) can be identified for an expenditure of only $80 (USD)/sample.

## Introduction

Genetic variants, particularly single nucleotide polymorphisms (SNPs), are the basis of genetics and enable study of the genetic mechanism underlying human diseases and agriculturally important traits. Recently, next generation sequencing (NGS) technology has enabled the discovery of hundreds of thousands of SNPs and validation by “parallel” sequencing with plummeting cost [1], [2]. Most NGS methods, such as genotyping by sequencing (GBS) [3], multiplexed shotgun genotyping (MSG) [4] and restriction site-association DNA sequencing (RAD-seq), depend on restriction enzymes to produce a reduced representation of a genome [5]. The GBS and MSG methods, which rely on low sequencing depth (<5×/site per individual, on average), are simple and cost-effective approaches for genotyping inbred populations in which the parental genotypes are either known or can be assigned probabilities. Accurate genotype calling, however, is difficult to achieve by low sequencing depth approaches for general outbred populations because of the high degree of heterogeneity and phase ambiguity in the haplotype.

The RAD-seq method, which sequences target regions deeply (>20×) and enables markers to be genotyped accurately for outbred populations, is expensive and labor-intensive for high-throughput SNP detection because of the high sequencing depths and complex library preparation protocol [6].

Human and some major farm animals (cattle, sheep and pig) are outbred populations of species with high level of heterogeneity. There are considerable differences in genome size and structure compared to the inbreeding plant species. So, the library preparation and the method of marker discovery and genotyping have its uniqueness. Apparently, flexible and cost-effective protocol is required that can be implemented in outbred populations.

In order to balance the cost and accuracy of genome-wide marker discovery and genotyping, we report a medium sequencing depth (5–20×/site per individual, on average) approach called genotyping by genome reducing and sequencing (GGRS) to address the challenge of genotyping an outbred population. The GGRS approach is mainly based on the simple procedure of library preparation and a marker discovery and genotyping pipeline. It is effortless and highly reproducible to reduce the genome complexity to ensure sufficient sequence coverage especially for species with large genomes for our GGRS approach. The goal of this article is to describe the approach and evaluate its efficiency and accuracy for high-density SNP discovery and genotyping in a outbreed population.

## Methods

### Library Preparation

#### (1) Procedure of library preparation

**Figure 1:**
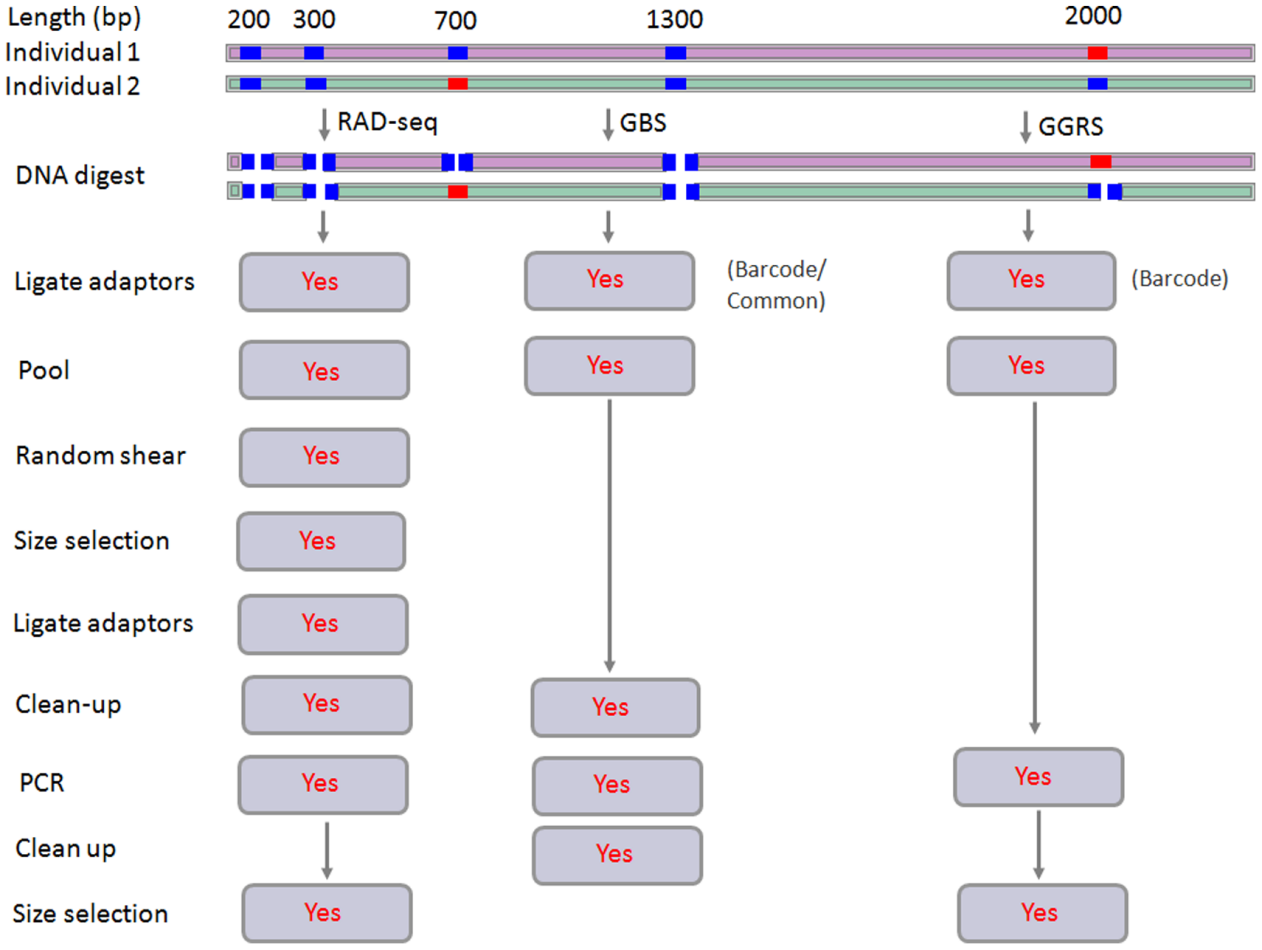
Comparison of RAD-seq, GBS and GGRS methods for library preparation. Blue block, an example of a genomic region containing restriction sites; red block, variation in the cut site at 2000 bases of individual 1 and 700 bases of individual 2 and is not cut by the restriction enzyme. Individuals 1 and 2 are light pink and light green, respectively. The red word Yes with gray shading shows that the step is necessary in the protocol. Both GBS and GGRS are simpler than RAD-seq; furthermore, GGRS discards two clean-up steps in GBS instead of size selection.

The procedure of library preparation for GGRS approach was improved based on RAD-seq and GBS to adapt genotyping for outbred populations. **Figure 1** illustrates the comparison of GGRS, RAD-seq and GBS methods for library preparation. Compared to RAD-seq, our protocol for the preparation of sequencing libraries includes a number of simplifications, including fewer gel-purification steps, no random shearing or end-repair of fragments and only one set of adapter-barcodes. This new and very simple library preparation protocol allows us to work with a small number of genomic DNAs and to reduce both labor intensity and cost.

Compared to GBS, our GGRS approach further improves the procedure to make it more suitable for use with an outbred population while keeping the simplicity, the rapidity and the reproducibility. Moreover, the GGRS approach includes three improvements: 1) fragments were selected by gel electrophoresbased on the genomic properties of an outbred population, which decreased cost, 2) one set of adapter-barcodes was designed to meet the requirements of depth and coverage to attain greater genotype accuracy, and 3) GGRS method merged removing-adapter steps and PCR clean-up steps into the last PCR gel-purification steps to reduce variation of fragment number between individuals.

#### (2) DNA sources

Approval by the Institutional Animal Care and Use Committee of Shanghai Jiao Tong University (contract no. 2011–0033) was given for all experimental procedures involving animals in the present study.

The GGRS procedure was initially established for a large genome pig species (about 2.8 Gbp). DNA samples were obtained from 36 Landrace pigs and 36 Large White pigs. High molecular weight genomic DNAs were extracted from ear tissue using the Multisource Genomic DNA extraction kit (Axygen Biotechnology (Hangzhou) Co., Ltd, China).

#### (3) Choosing restriction enzymes (REs)

According to the different levels of linkage disequilibrium (LD) between breeds from different regions, haploblocks are up to 10 kb in Chinese breeds and up to 400 kb in European breeds [7]. We need to identify markers that cover around 2.8×10^5^ genomic locations to enable GWAS in Chinese breeds. However, as a matter of fact, about 5.6×10^5^ genomic location were covered according to sequencing method of paried-end. Restriction enzyme *Ava*II, which recognizes the degenerate 5 bp DNA sequence GGWCC where W denotes A or T, was selected to cut the porcine genome frequently and avoid repetitive elements. The resulting fragments by digestion with *Ava*II have a 5' sticky end of 3 overhanging bases (GWC) of the top strand. The GWC motif is complementary to the 3' overhang of 3 bases (CWG) of the bottom strand of a set of 72 adapter-barcodes. In the light of digestion of the reference genome by *Ava*II *in silico*, resulting fragments of 200–300 bp showed an anticipated coverage of ∼2.0% of the reference genome and ∼2.8×10^5^ unique fragments each with 200 bases sequenced by paired-end, i.e. ∼5.6×10^7^ bases ideally aligned with the reference genome. We selected the range of 300–400 bp as sequencing fragments because the primers were added to the two ends of fragments by PCR.

#### (4) Adapter-barcode design

Only one type of adapters following the standard Illumina sequence was used for paired-end DNA libraries with a set of barcodes of 4–8 base modifications (**Table S1**) on the 3' end of the plus strand and on the 5' end of the minus strand. The sequences complementary to the barcode and the generated overhang bases GWC were added by *Ava*II. The plus and minus oligonucleotide strands were: 5′ACACTCTTTCCCTACACGACGCTCTTCCGATCTXXXXX3′,5′GWCYYYYYAGATCGGAAGAGCGGTTCAGCAGGAATGCCGAG3′

where XXXXX and YYYYY denote the barcode and the reverse barcode complementary sequences, respectively. When annealed, the plus and minus strand oligonucleotides formed the double-strand divergent Y formation. The adapter-barcodes were ligated on the two ends of digested fragments by complementary overhang sequences (**Figure S1**). The barcode length diversity of 4–8 bases can avoid to sequence the same base at the same site of total reads in the same sequencing cycle and is effective in reducing the error rate of base calling. The major reason is that generating an invariant GWC sequence at the first 3 bases of all reads with the same length barcode probably caused sequencing phasing error, and base calling errors in subsequent analysis [3].

Stock solutions of 100 μM plus and minus strand adapters were made in 10 mM Tris–HCl (pH 7.8). Solutions of the appropriate adapter pairs were mixed 1∶1 (v/v) to give a final concentration of 50 μM, and then the annealing reaction was run in a ABi Veriti thermocycler. The mixture was heated at 95°C for 2 min, and then ramped down to 25°C at a rate of 0.1°C/s, kept at 25°C for 30 min and naturally cooled to 4°C. The 5′ end phosphorylation of the annealed mixture was performed in a thermocycler. The 10.0 μl reaction mixture, which included 1.0 μl of 10×T4 PNK buffer, 1.0 μl of 10 mM ATP, 0.1 pM (about 2.5 μg) annealed mixture and 10 U of T4 PNK (NEB co., USA) was incubated at 37°C for 1 h. After heating inactivated at 65°C for 20 min, the phosphorylated adapter was diluted to a concentration of 2 ng/μl (0.07 pM/μl) before ligating reaction.

#### (5) Preparation for sequencing libraries

We diluted genomic DNA to ∼50 ng/μl (Quantified by PicoGreen, Promega, USA) and transferred 100 ng of DNA from each sample into 72- well PCR plates. The DNA was digested with 10 U of restriction enzyme *Ava*II (NEB Co., USA) in a volume of 10 μl at 37°C for 6 h, followed by inactivation by heating at 65°C for 20 min and then naturally allowed to cool to 4°C. A unique adapter-barcode sequence was ligated to each individual's sticky end by 200 cohesive end unites of T4 DNA ligase (NEB Co., USA) at 22°C for 2 h, then the ligase was heated at 65°C for 30 min and the mixtures of each ligation reaction were pooled. The pooling libraries were prepared from 5 independently parallel PCR-enriched in a final volume of 50 μl containing 25 μl of 2× phusion PCR Mastermix (Laifeng Biotech Co., Ltd, China), 2 μl of pooled DNA fragments, 2 μl of 10 pM primer 1.1 and 2 μl of 10 pM primer 2.1.The pairs of PCR primers followed the standard Illumina sequences:

Primer 1.1

5′AATGATACGGCGACCACCGAGATCTACACTCTTTCCCTACACGACGCTCTTCCGATCT

Primer 2.15′CAAGCAGAAGACGGCATACGAGATCGGTCTCGGCATTCCTGCTGAACCGCTCTTCCGATCT

The primer pairs contained primer sequences and complementary oligonucleotide sequences attached to the flow cell. This design was essential to sequence by paired-end using only a set of adapter-barcodes.

The thermal cycling protocol for sequencing libraries preparation was: 95°C for 5 min, then 20 cycles of 95°C for 30, 65°C for 30, 72°C for 30, and a final elongation step at 72°C for 10 min. Each of five separate libraries was independently purified from PCR mixture using a DNA gel extraction kit (Axygen Biotech (Hangzhou) Co., Ltd, China) and then five separate libraies were mixed into one sequencing libraries. The quality of sequencing libraries was evaluated by an Agilent 2100 bioanalyzer. Sequencing libraries were used for next-generation sequencing if the fragments were in the range 300 to 400 bp. If not, the sequencing libraries were reconstructed using the protocol described above. A 72-plex sequencing libraries for each flow cell lane was sequenced by an Illumina Hiseq2000 instrument with a paired-end (2×100 bp) pattern, and the sequencing process is given in detail by the manufacturer (Illumina).

### Genome-wide Marker Discovery and Genotyping

#### (1) Filtering raw sequencing data

We filtered sequences according to several rules for subsequent analysis [3]: a) carrying one of the barcodes to distinguish individuals; b) sequences with the first 3 bases of the restriction motif GGWCC; c) no adapter/adapter dimer or polymer; d) no-calling bases of the first 80 bases per reads.

#### (2) Aligning reads to the reference genome

In this study, although we used the paired-end method for sequencing, we aligned filtered reads with the pig reference genome (SGSC Sscrofa9.2) by the single-end mapping method because of the imperfection and breed specificity of the reference genome. We aligned reads to reference genome using Burrows-Wheeler Aligner (BWA) with the default settings [8] by three steps: 1) mapping all filtered reads to the reference sequence; 2) dividing remaining single reads into two or three shorter reads according to the restriction motif sequences and aligning them individually to the reference genome; 3) querying the remaining reads by sliding window method to make sure that we can make use of the incomplete reference panel.

#### (3) Calling SNP and determining the genotype of an individual

The successfully aligned reads were simultaneously mapped to the pig reference genome using SAMtools with default settings to discovery SNPs [9]. In brief, for a genotype G, Bayes' formula was used to calculate the posterior probability of genotype G in conjunction with a genotype prior and a genotype likelihood. The priors were improved by analyzing multiple individuals. The method for calculating genotype likelihood and other details were described in Mathematical Notes on SAMtools Algorithms by Heng Li (http://www.broadinstitute.org/gatk/media/docs/Samtools.pdf).

#### (4)  = Genotype imputation

The missing genotype was imputed using iBLUP software developed by our group. The iBLUP software is available at http://klab.sjtu.edu.cn/iBLUP/.

## Results

### Reads Quality and Quantity

**Figure 2:**
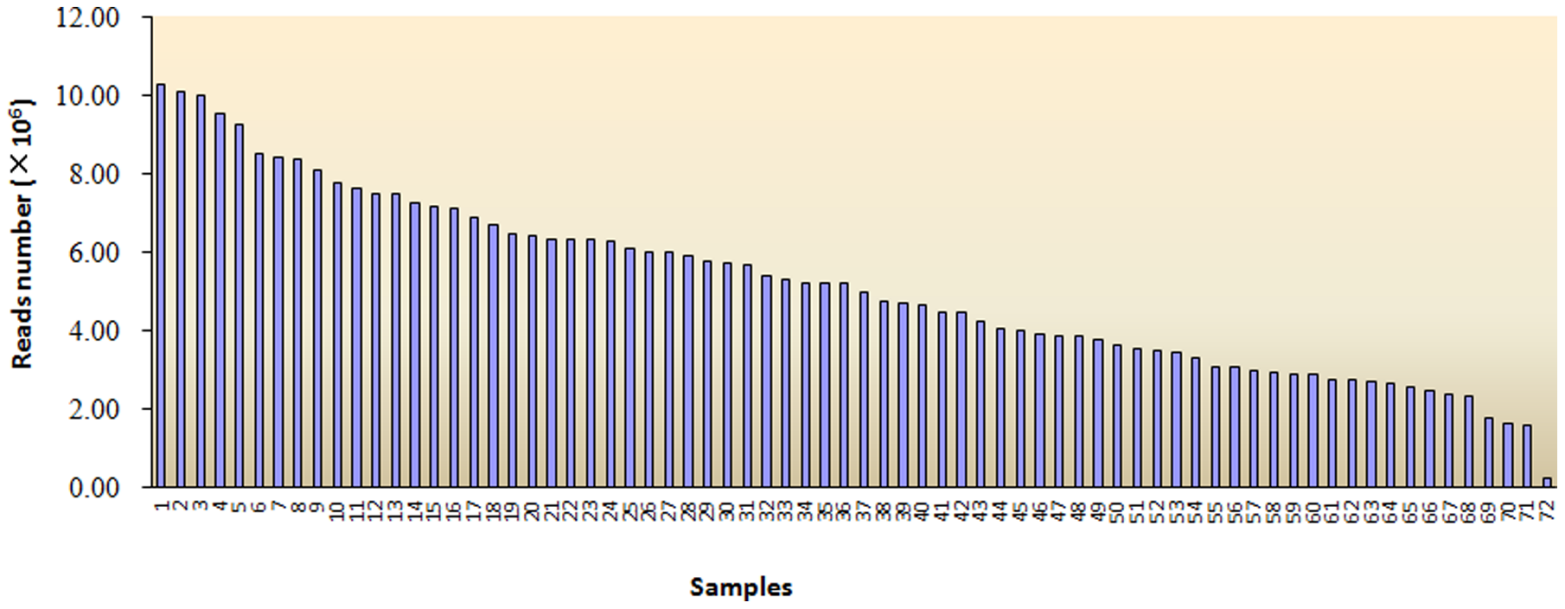
The distribution of high quality reads across 72 individuals.

Sequences were collected in lanes of a single flow cell at 72-plex from a pig outbred population, a genetically diverse and large genome species. A total of 380,971,530 raw reads were generated in a flow-cell lane of an Illumina High-seq 2000 sequencer for the pig population. 361,611,915 (94.9%) reads complying with the filtering rules were high-quality reads. Of which, the maximum and minimum of reads are 10,077,526 and 1,570,923, respectively. The average reads number was 5,022,387. However, one particular individual with 229,575 reads may be due to random error (**Figure** **2**). The variation of reads number between individuals was considerably lower (cv was about 43%) than that achieved by the MSG method (cv was about 89%) and the same as that achieved by the GBS method (cv was about 43%).

The base average quality score, a base identifying probability, was at least 20 (error rate of base calling of 1 in 100), in which the average quality score of the first 65 bp was at least 30 (error rate of base call of 1 in 1000) (**Figure S2**). The first three bases had poor quality scores, then which gradually increased and decreased along the reads length from 5' end to 3' end. The fact mainly attributed to the algorithm of base caller from illumina sequencing platform. The higher quality score and longer sequence per reads have advantages of true SNP discovery by validly mapping to reference genome sequence, which was crucial for accurate genotyping by directly sequencing per individual. The sequecing data has been deposited in NIH Short Read Archive(Accession number: SRX288441).

### Sample Representation

**Figure 3:**
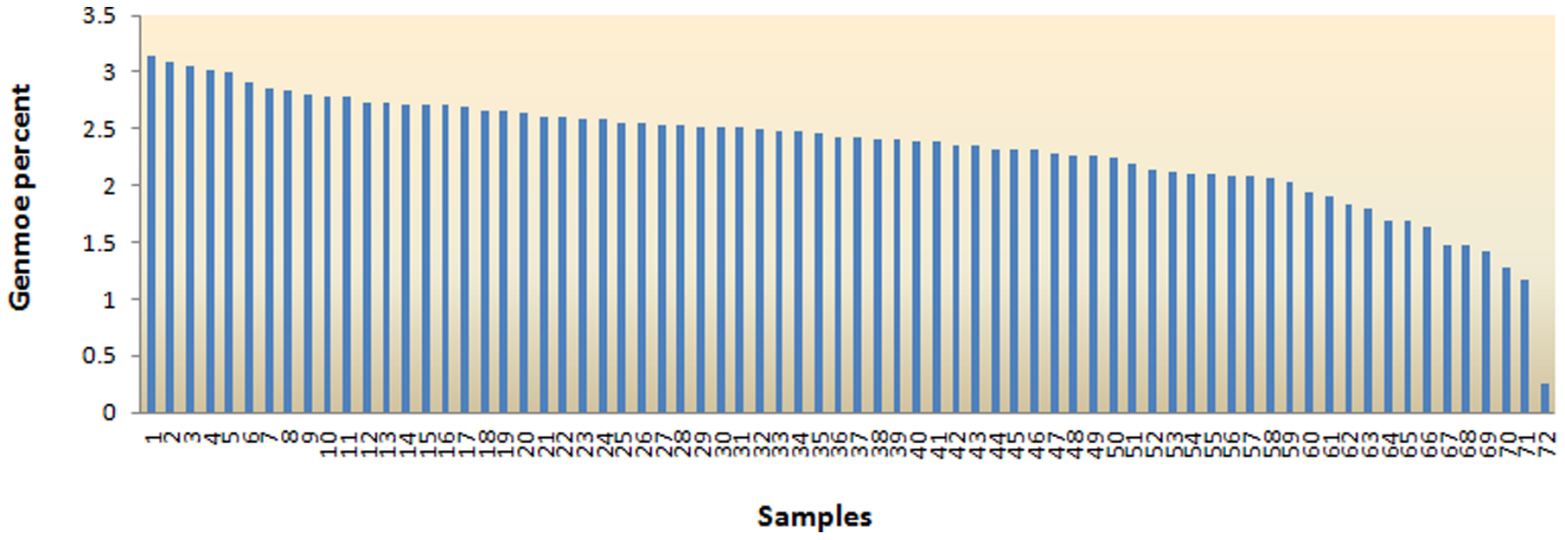
The genome percent of high quality non-redundant reads across 72 individuals.

The sequencing results demonstrated that our GGRS pipeline yielded high-quality scores and a small variation of reads number between individuals by reducing genome complexity. Of “high quality” reads, 318,218,485(88%) reads were aligned with pig reference genomic sequences using BWA with default settings. The majority of no-aligning reads to reference panel represented pig genome sequences by Basic Local Alignment Tool (BLAST) of National Center for Biotechnology Information (NCBI) nt database using default settings (data not shown), which showed that a large number of gaps existed in reference sequences of using version. These sequences were used to produce consensus sequences to slightly supply reference genome. In general, assigned reads of each individual uniformly distributed across chromosomes, although there were few regional variations.The density distribution of high quality non-redundant reads across autosomes and X chromosome were showed in **Figure S3**. On average, the reads density was ∼5.4 reads/10 kb. The total site number of all reads aligned to the reference panel was 604,379 with average sequencing depth of 5.97× and coverage of 2.16%. Of which, the minimum and maximum were respectively 171,472 and 447,051 except for above-mentioned special individual. The coefficient of variation of the site number between individuals was about 21% (**Table S2**). The total genome location number of all reads aligned to reference panel roughly met with the experiment design. On average, the sequencing coverage accounted for about 2.33% of genome content (**Figure 3**).

### Mapping and SNP Validation

In order to reduce the false positive SNPs, we taked the following three criteria: 1) the reads/site/individual aligned to build a reliable sequence reads was more than 5× [10]; 2) the minimum number of genotyped individuals was not less than 15%; 3) the minor allele frequency was not less than 5%.

In the present pig sequencing experiment, 71,072 SNPs were discovered and 46% missing genotypes were imputed using the iBLUP program. On average, the density of putative SNPs across chromosomes was ∼0.33 SNPs/10 kb. The maximum and minimum were 0.79 on chromosome 6 and 0.19 on X chromosome, respectively (Figure S4). The density distribution of putative SNPs across chromosomes except chromosome 6 was showed to be approximately uniform. The calling SNPs can be downloaded at http://klab.sjtu.edu.cn/GGRS/.

## Discussion

For large genome outbred species, reducing genome complexity, optimizing barcode and simple procedure are key points for sequence libraries preparation.

The step of selecting fragments appears to be necessary for outbred species for the aim of obtaining sufficient sequencing depth for calling more accurate SNPs and genotyping. Currently, most of available genome reducing and sequencing methods are based on restriction enzymes (REs) [3], [4], [5]. The key points for selection of appropriate REs are to avoid frequently cutting repetitive elements and to generate ranges with fragment sizes suitable for sequencing coverage across genome and sequencing depth. The results of several digesting experiments using a few REs showed that *Ava*II was a relatively perfect REs for the pig sequencing experiment (data not shown). To compare with GBS method, we selected digesting fragments of ≥200 bp by gel purification to sequence because digesting fragments of <200 bp would be partly or completely sequenced twice or more by paried-end using Hiseq 2000 platform. Although these repeating sequencing fragments improved the accuracy of SNP calling, the method probably increased the variation of reads and decreased the efficient utilization rate of reads. Some next-generation sequencing methods including GBS were excellent to genome-wide genotyping for inbred population with low coverage and small genome animals with low cost. For outbred populations, using these methods to accurately genotype was difficult to achieve with low depth and coverage because of the high extent of heterogeneity and phase ambiguity in the haplotype. The genotyping results of pig experiment showed that GGRS were qualified for outbred population. Furthermore, for other outbred animals with smaller genome, lower heterogeneity or more information than pig (such as chicken), if lower genome coverage and sequencing depth are desired, the procedure can be modified using different restriction enzymes or altering sequencing fragments and thus GGRS pipeline is generally applicable.

Our GGRS procedure employed one set of adaptors. Two ends of digested resulting fragments were ligated to identical barcode-adaptor, instead of one end ligated common-adaptor and the other end ligated barcode-adaptor. This barcode-adaptors design was beneficial for outbreed population by increasing fragments consistency between individuals. The GGRS is appropriate for parallel genotyping of large number of samples than existing RAD protocols because of the simpler procedure, and the optimized protocol for libraries preparation is helpful for saving cost and labor. Moreover, the simplified protocol allows us to use small amounts of DNA for libraries preparation (about 100 ng, even lower) that is important for studying rare materials.

Recently, a streamlined restriction site-associated DNA genotyping method called 2b-RAD has been published [11]. The choice of restriction enzyme type of 2b-RAD is a good idea that produced even coverage across genome. In addition, this method can change marker density by modifying the overhang bases of adaptors. However, the flexibility of changing marker density is not enough because of the restriction property of type IIB restriction enzyme. Further, the lengths of the restriction fragments are uniform and short that cannot make full use of the sequencing performance of Hiseq2000 platform with 2×100 bp of paried-end. Compared to 2b-RAD, our methods can generate more abundant data in one lane to decrease cost, and longer reads improve the accuracy of aligning with reference genome although the advantage of 2b-RAD method lies in the well-distributed of markers. Both 2b-RAD and GGRS approaches can perform *de novo* analysis for the outbred species lacking an assembled genome sequence easily. In such situation, clustering reads can be regarded as the reference sequence.

Our GGRS pipeline can process 504 samples each run (72 samples/lane ×7 lanes/flow-cell, one lane as control) and >70, 000 pig SNPs can be identified in a short time for an expenditure of $80 (USD)/sample. Up to 288 samples each lane (2016 samples/flow-cell) will possibly be sequenced as along with increasing reads density of upgrading Hiseq 2000 sequencer. These improvements will accelerate the reduction of the genotyping cost to <$20 (USD)/sample.

## Supporting Information

Figure S1 **Ligation fragment generation.** (A) Plus strand and minus strand were annealed to “Y” formation adapter-barcode; (B) Genomic DNAs were digested to DNA fragments with 5′ overhang “GWC” by AvaII restriction enzyme; (C) The formed adapter-barcodes were ligated on the two ends of digested fragments by overhang complement sequences.
(TIF)

Figure S2 **The average quality score of the sequencing data.** The base-identifying probability was at least 20 (error rate of base calling of 1/100) and at least 30 in the first 65 bp (error rate of base call of 1/1000).
(TIF)

Figure S3 **The distribution of high quality non-redundant reads across autosomes and the X chromosome.**
(TIF)

Figure S4 **The density distribution of SNPs on chromosomes.**
(TIF)

Table S1 **GGRS barcodes sequences.**
(DOC)

Table S2 **Genome location number.**
(XLS)
