# Supplementary figures and images for: Genotyping by Genome Reducing and Sequencing for Outbred Animals
Source: PLoS One. 2013 Jul 18;8(7):e67500. doi: 10.1371/journal.pone.0067500 (PMC3715491; doi:10.1371/journal.pone.0067500)

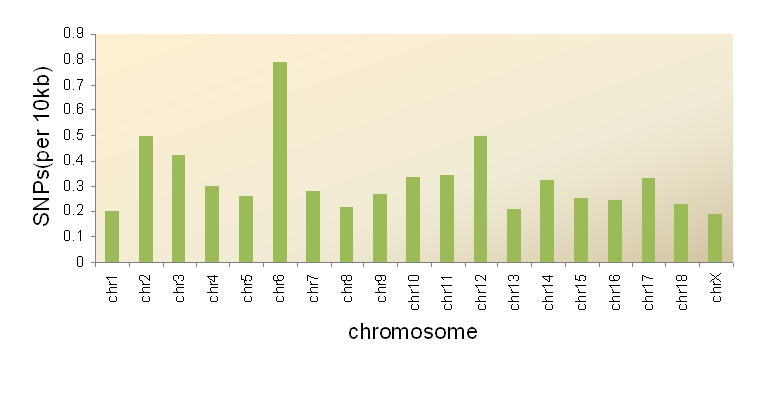

Supplement: Figure S4 — The density distribution of SNPs on chromosomes. (TIF) [file pone.0067500.s004.tif]
