# Supplementary material for: Genotyping by Genome Reducing and Sequencing for Outbred Animals
Source: PLoS One. 2013 Jul 18;8(7):e67500. doi: 10.1371/journal.pone.0067500 (PMC3715491; doi:10.1371/journal.pone.0067500)
Supplement: Table S1 — GGRS barcodes sequences. (DOC) [file pone.0067500.s005.doc]

**Table S1 GGRS barcodes sequences**

| AACT | CCTAC | TTCTC | AATATGC | ATTAATT | CTTGCTT |
| --- | --- | --- | --- | --- | --- |
| CAGA | CTGTA | ACCTAA | ATGAAAC | ATTGA | GAACTTC |
| CGAT | GAGGA | ATATGT | CGGTAGA | CATCGT | GCTGTGGA |
| GATC | GGAAC | CCAGCT | GTCGATT | CCACAA | GGACCTA |
| GCGT | GGTGT | GAGATA | TCGAAGA | CCGGATAT | GGATTGGT |
| GTAA | GTATT | GCCAGT | AAAAGTT | CGCGGAGA | GTTGAA |
| TCAC | GTCAA | GCTCTA | AACCGAGA | CGCTGAT | TAGGAA |
| TGCA | TAATA | GGTTGT | ACGACTAC | CGCTT | TAGGCCAT |
| ACAAA | TACAT | TAACGA | ACGTGTT | CGTGTGGT | TATTTTT |
| AGCCC | TCACC | TGGCTA | AGGC | CTAGC | TCTCAGTC |
| AGGAT | TCGTT | TTCAGA | AGTGGA | CTATTA | TCTGTGA |
| CATCT | TGCGA | AACGCCT | ATGCCT | CTCC | TTCCTGGA |
